# A Randomized Trial of Deep Brain Stimulation to the Subcallosal Cingulate and Nucleus Accumbens in Patients with Treatment-Refractory, Chronic, and Severe Anorexia Nervosa: Initial Results at 6 Months of Follow Up

**DOI:** 10.3390/jcm9061946

**Published:** 2020-06-22

**Authors:** Gloria Villalba Martínez, Azucena Justicia, Purificación Salgado, José María Ginés, Rocío Guardiola, Carlos Cedrón, María Polo, Ignacio Delgado-Martínez, Santiago Medrano, Rosa María Manero, Gerardo Conesa, Gustavo Faus, Antoni Grau, Matilde Elices, Víctor Pérez

**Affiliations:** 1 Department of Neurosurgery, Hospital del Mar, 08003 Barcelona, Spain; gloriavillabamartinez@gmail.com (G.V.M.); idelgadom@parcdesalutmar.cat (I.D.-M.); gconesa@parcdesalutmar.cat (G.C.); 2 Department of Psychiatry and Forensic Medicine, Universitat Autònoma de Barcelona, Cerdanyola del Vallès, 08193 Barcelona, Spain; vperezsola@parcdesalutmar.cat; 3 Hospital del Mar Medical Research Institute (IMIM), 08003 Barcelona, Spain; ajusticia@imim.es; 4 Institut de Neuropsiquiatria i Adiccions (INAD), Hospital del Mar, 08003 Barcelona, Spain; psalgado@parcdesalutmar.cat (P.S.); jgines@parcdesalutmar.cat (J.M.G.); rguardiola@parcdesalutmar.cat (R.G.); ccedron@parcdesalutmar.cat (C.C.); mpolo@parcdesalutmar.cat (M.P.); 5 Centro de Investigación Biomédica en Red de Salud Mental (CIBERSAM), 28029 Madrid, Spain; 6 Department of Radiology, Hospital del Mar, 08003 Barcelona, Spain; santiago.medrano@upf.edu; 7 Department of Neurology, Hospital del Mar, 08003 Barcelona, Spain; rmanero@parcdesalutmar.cat; 8 Department of Surgery, Universitat Autònoma de Barcelona, Cerdanyola del Vallès, 08193 Barcelona, Spain; 9 ITA, Mental Health Specialists, 08036 Barcelona, Spain; gfaus@ita.com (G.F.); agrau@ita.com (A.G.)

**Keywords:** anorexia nervosa, deep brain stimulation, psychosurgery, clinical trial, subcallosal cingulate, nucleus accumbens, body mass index

## Abstract

Background: The main objective of this study was to assess the safety and efficacy of deep brain stimulation (DBS) in patients with severe anorexia nervosa (AN). Methods: Eight participants received active DBS to the subcallosal cingulate (SCC) or nucleus accumbens (NAcc) depending on comorbidities (affective or anxiety disorders, respectively) and type of AN. The primary outcome measure was body mass index (BMI). Results: Overall, we found no significant difference (*p* = 0.84) between mean preoperative and postoperative (month 6) BMI. A BMI reference value (BMI-RV) was calculated. In patients that received preoperative inpatient care to raise the BMI, the BMI-RV was defined as the mean BMI value in the 12 months prior to surgery. In patients that did not require inpatient care, the BMI-RV was defined as the mean BMI in the 3-month period before surgery. This value was compared to the postoperative BMI (month 6), revealing a significant increase (*p* = 0.02). After 6 months of DBS, five participants showed an increase of ≥10% in the BMI-RV. Quality of life was improved (*p* = 0.03). Three cases presented cutaneous complications. Conclusion: DBS may be effective for some patients with severe AN. Cutaneous complications were observed. Longer term data are needed.

## 1. Introduction

Anorexia nervosa (AN) is a psychiatric disorder with an estimated prevalence of 0.7–3%. It primarily affects females and is usually diagnosed in adolescence and young adulthood [1,2]. AN is a life-threatening illness that can have a devastating impact on patients and their family [2,3]. The Diagnostic and Statistical Manual of Mental Disorders (DSM-5) criteria define two subtypes of AN: the restricting type and the binge-eating/purging type, with the latter type (purging) having a worse prognosis. Treatment of AN involves a combination of nutritional, pharmacological, psychological, and family interventions, all of which aim to restore normal weight, alter behavioural patterns, and address associated psychological issues. However, the optimal treatment for AN remains unclear and controversial [4,5].

Clear criteria to determine treatment refractoriness has not been fully established yet for this complex illness. Refractoriness in AN is currently defined as a failure to respond to repeated interventions over an extended time period (5–10 years), with recovery considered unlikely or, at best, limited in patients who have had AN for more than 10 years [3,6]. This condition is believed to be multifactorial, including neurobiological, environmental, and genetic factors, among others. Several studies seem to agree that AN is primarily caused by neurobiological alterations provoked by underlying dysfunction in the brain circuits. Although numerous models have been proposed to explain this dysfunction, most researchers agree that limbic system alterations are likely the main cause [7,8,9,10,11,12,13]. It has also been suggested that AN is, at least partially, maintained by dysfunctional activity in key neuroanatomic circuits [14,15,16,17], primarily those related to the modulation of reward and motivation, such as the mesolimbic cortex and the striatum [8,12].

Some authors have suggested that brain areas involved in the cognitive control of appetite (dorsolateral prefrontal and the parietal cortex) could also be involved in the pathophysiology of AN [10,13]. Morphological and functional studies in patients with AN have shown alterations in insular activity and in the prefrontal cortex, orbitofrontal, temporal, parietal, anterior cingulate, and ventral striatum (nucleus accumbens, NAcc) [18,19,20]. The available evidence suggests that the two most relevant targets for the surgical treatment of AN appear to be the NAcc [21] and the subcallosal cingulate (SCC), mainly due to the substantial involvement of these two structures in the reward circuits, but also because these two areas serve as communication links between the limbic and cortical systems [7,9].

Deep brain stimulation (DBS) is a surgical technique with a long history in the treatment of movement disorders such as Parkinson’s disease, dystonia, and essential tremor, offering good outcomes with only minimal complications [10,22,23]. In recent decades, this technique has also been used to treat mental disorders such as obsessive-compulsive disorder (OCD), major depressive disorder (MDD), and schizophrenia [24,25]. However, OCD is the only mental disorder for which DBS is currently approved by both the United States Food and Drug Administration (FDA) and by the European Union (CE Marking certification) [24,26,27]. In other mental disorders, DBS has only been performed in the context of clinical trials or for compassionate use.

Although the precise mechanism of action of DBS remains unclear, several models and hypotheses have been proposed. Electrophysiological studies suggest that the effect of DBS depends on the type of stimulated brain tissue (e.g., grey or white matter) and on the type of fibers involved in the stimulation [13,28,29]. In addition, DBS is believed to alter neuronal discharge patterns in the target area (jamming effect). These different mechanisms may thus combine both inhibitory and excitatory processes, which could act simultaneously. Even though DBS is applied locally to a specific brain area, both focal and distal effects have been reported [29].

Experience with DBS in patients with AN is limited. To date, a total of 26 women with AN, with variable clinical characteristics (e.g., severity, chronicity) have participated in clinical studies. Moreover, those studies targeted different brain structures. McLaughlin et al. reported a case of a patient with comorbid AN and OCD (body mass index (BMI): 18.5) whose condition improved slightly after DBS to the ventral striatum [30]. Wang and colleagues described two cases of adolescent patients with AN who received bilateral DBS to the NAcc, with no complications; over the course of 12-month follow up, both patients in that study successfully reached a normal BMI and also showed improvements in psychopathological symptoms and quality of life. Wu et. al. [31] also reported results of a case series of four adolescents with AN in which DBS was applied to the NAcc. In all four cases, psychological symptoms improved and body weight increased by up to 65%, without complications over a follow-up period that ranged from 9 to 50 months. In that same year (2013), Lipsman et al. reported results from a phase 1 pilot trial in which DBS was applied to the subcallosal cingulate in 6 patients with AN [32]. At nine months of follow up, half of the patients showed a response to treatment (BMI above baseline values); in addition, four patients presented improved psychometric assessments. Israel et al. published a case report of a patient with MDD and comorbid AN, who received DBS to the SCC (unilateral, right side, intermittent). The results were good and the patient successfully maintained BMI = 19.6 for more than 30 months [33]. In 2017, the same research group reported the results of a 12-month clinical trial involving 16 patients with AN (including the six patients from the original study) who received DBS to the SCC. Mean BMI values and psychometric assessments improved in all 16 patients over time; moreover, a flurodeoxyglucose–positron-emission tomography (FDG-PET) scan showed metabolic changes in the brain after six months of DBS. However, several adverse events were reported, including one event of each of the following: air embolism, seizure, skin infection, worsening in mood, and intraoperative panic attack, and five of the patients also experienced pain [34]. Blomstedt and colleagues published a case report of a patient with MDD and comorbid AN in whom DBS was administered to the bed nucleus of the stria terminalis (BNST). Although the procedure did not significantly change the BMI, it did improve the patient’s anxiety about food and eating [35]. Finally, the most recently published study was reported by Manuelli et al. (in 2019), who described the case of a patient with moderate AN who underwent DBS to the BNST. In that patient, BMI, core AN symptoms, and nutritional status all improved at six months of follow up [36].

Together, the limited evidence for DBS as a treatment for AN suggests that DBS appears to be a safe and effective treatment. However, due to the heterogeneity of this disorder, and the difficulty of recruiting patients to participate in clinical trials and studies, it is difficult to make definitive conclusions about the efficacy of DBS, the optimal target site, and the clinical and radiological variables that determine response. In this context, the main aim of the present clinical trial was to assess the efficacy and safety of DBS applied to two different targets (SCC and NAcc) to treat patients with chronic, severe, refractory AN. This study has been divided into three distinctive phases. Phase I involved the selection of participants and preoperative procedures. Phase II involved the surgical procedure itself, including a 6-month period of active stimulation. In phase III, patients considered responders to the DBS implant were randomized to one of two arms (ON/OFF or OFF/ON), while non-responders were not randomized, rather, they continued to be assessed monthly throughout the 12-month follow-up period. As phase III is currently ongoing, the current report presents data on phase I (preoperative) and phase II (6 months follow up).

## 2. Materials and Methods

### 2.1. Participants and Setting

A total of eight participants diagnosed with chronic, severe, refractory AN were included in this trial over a one-year period, which was conducted jointly by the Departments of Psychiatry and the Surgery Department of the Hospital del Mar in Barcelona, Spain, a tertiary care university hospital. Participants were recruited from collaborating sites around Spain, including the *Eating Disorders Institute, Mental Health Specialists* (in Spanish, *ITA Especialistas en Salud Mental)*, a national network of hospitals and treatment centers across Spain providing specialized care for patients with eating disorders.

The inclusion criteria were as follows: age: 18–60 years, clinical diagnosis of any type of AN (DSM-5 criteria), duration of AN > 10 years, treatment-resistant AN, defined as follows: (a) lack of response to ≥3 voluntary intensive treatments (full or partial hospitalization) or (b) clinical worsening and unwillingness to receive any further treatment, including ≥2 hospital admissions for involuntary feeding, preoperative BMI between 13 and 15.99 (patients with BMI values outside this range could be included on case-by-case basis), and capacity to fully understand the study and to provide informed consent. Exclusion criteria: current or past psychotic episode, comorbid neurological illness, drug abuse in the last year, contraindications to undergo magnetic resonance imaging (MRI) or DBS, any medical condition involving a risk for the surgical procedure, and pregnancy.

Written informed consent was obtained from all participants before proceeding with any intervention. The study was performed according to the ethical standards stated in the Declaration of Helsinki and subsequent updates. The study was approved by the Ethics Committee of the Parc de Salut (Barcelona, Spain, approval number: 2016/6813/I). Trial registration: Clinicaltrials.gov: NCT03168893.

### 2.2. Design and Procedure

This is a randomized, double-blind, controlled crossover clinical trial consisting of two consecutive 6-month phases (total duration: 12 months). Target selection was based on the presence of comorbidities with other psychiatric conditions and the type of AN. Patients whose predominant comorbidity was an affective disorder received DBS to the SCC, while patients whose predominant comorbidity was an anxiety disorder received DBS to the NAcc. The predominant comorbidity was determined by a clinical psychiatrist based on the MINI International Neuropsychiatric Interview scores and a comprehensive clinical interview. Each case was then reviewed by the other members of the clinical research team (a psychologist and a psychiatrist) to ensure their assessments for each comorbidity were consistent. For patients that did not show any clear predominant comorbidity, the target was selected based on the type of AN: patients with binge-eating/purgative AN received DBS to the NAcc, and patients with restrictive AN received DBS to the SCC. If a patient presented criteria for both targets, the target that corresponded to the most severe comorbidity was selected.

Phase I of the study consisted of the patient recruitment and selection. Potential candidates were interviewed by a member of the research team (psychiatrist). After this initial screening, an independent, external psychiatrist confirmed that the patient met all inclusion criteria. Patients were required to present normal results on all of the following preoperative tests: chest x-ray, electrocardiogram, and anesthesia tolerance test. Minimal alterations in blood test results were allowed, provided that these were normalized prior to surgery. The optimal pre-operative BMI was set at ≥13; in cases with a BMI < 13, the participant was admitted to the inpatient ward to raise the BMI to meet the minimum threshold. A 1.5 T MRI with diffusion tensor imaging (DTI) was performed pre-operatively. Before study inclusion, the participants’ BMI data in the previous year was registered (lowest, highest, and mean).

Phase II involved the surgical procedure and six-month follow-up. The DBS system was implanted using robotic stereotactic assistance (ROSA; Zimmer Biomet, Inc. Montpellier, France). The surgical procedure was performed under general anesthesia and divided into three steps: (1) fiducial markers were inserted followed by an intraoperative computerized tomography (CT) scan, (2) the ROSA robotic arm was prepared and electrodes (Infinity; Abbott Inc., Saint Paul, MN, USA) were placed on the selected target through two trephine holes, at the frontal level, bilaterally. The Infinity electrodes (Abbott Inc., Saint Paul, MN, USA) are directional electrodes with four contacts, 1.5 mm in diameter, with 1.5 mm spacing between contacts, with an inactive distal part, and (3) finally, a pulse generator was implanted subfascially at the right abdominal area and connected to the electrodes. A postoperative cranial CT scan was performed and fused with the preoperative MRI CT scan. Correct placement of the electrode contact points was verified. Next, monopolar stimulation was performed. The stimulation started at 3.5 milliamperes (MA) and was increased according to patient response. The frequency (130 Hz), pulse amplitude (90 micros), and contact were set to remain constant throughout the trial.

Stimulation began 24 hours after surgery and participants were discharged 72 hours after the intervention. At 10 days, an initial postoperative assessment was performed by the psychiatrist and neurosurgeon at the outpatient clinic, and subsequent evaluations were scheduled to be performed monthly. All participants were provided with contact details (telephone/email) for the surgeon and co-primary investigator to communicate any adverse events over the course of the trial.

**Figure 1:**
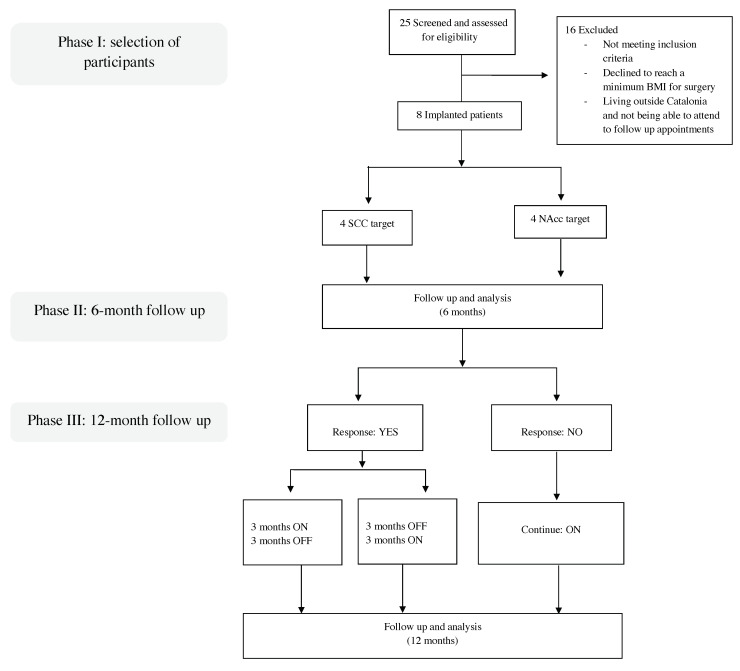
Participants’ selection and pathway for the randomized crossover controlled clinical trial. Here, results are presented including the follow-up analysis at 6 months.

A third and final phase (phase III) of this trial is still ongoing. Consequently, the present report focuses on describing phase I and on presenting the data from phase II (6-months follow up). A flowchart of the complete study design is shown in Figure 1.

### 2.3. Measures

A wide range of variables were collected, including sociodemographic characteristics of the sample and a complete clinical history.

Primary outcome: 

The primary outcome measure was BMI. Anthropometric measures (weight and BMI calculation) were collected at baseline, immediately before surgery, and monthly thereafter.

Secondary outcomes:

Secondary outcome measures include scores on a range of instruments described below.

Clinical outcome measures:

- The Mini-International Neuropsychiatric Interview (MINI) [37], a short diagnostic structured interview.
- The Hamilton Depression Rating Scale (HAMD_17_) [38], designed to assess depressive symptoms. Each item on the questionnaire is scored on a 3- or 5-point scale, depending on the item. The original version contains 17 items (HAMD_17_).
- Hamilton Anxiety Rating Scale (HAM-A) [39], a 14-item scale designed to rate the severity of anxiety symptoms. Each item contains a group of symptoms rated on a scale of 0–4, with 4 being the most severe.
- The Yale-Brown Obsessive Compulsive Scale (Y-BOCS) [40], a 10-item scale designed to measure the severity of illness in patients with obsessive-compulsive disorder, with a range of severity and types of obsessive-compulsive symptoms. Each item is rated from 0 (no symptoms) to 4 (extreme symptoms), with separate subtotals for severity of obsessions and compulsions.
- The Yale-Brown-Cornell Eating Disorders Scale (YBC-EDS) [41] is a semi-structured interview containing 8 items to assess the nature and severity of preoccupations and rituals related to the eating disorder.
- The Multidimensional Assessment of Interoceptive Awareness (MAIA) [42] is an 8-scale state-trait self-report questionnaire containing 32 items to measure multiple dimensions of interoception.
- Gardner Assessment of Body-Image [43] is a set of schematic contour scales to assess body disturbances.
- Barratt Impulsiveness Scale [44] is a 30-item self-report measure of impulsive personality traits.
- The Short Form Health Survey (SF-36) [45] is a 36-item instrument to assess quality of life.

Neuroimaging:

A brain MRI (1.5 T + DTI, 60 directions) was performed. Fractional anisotropy (FA), mean diffusivity (MD), axial diffusion (AD), radial diffusion (RD), and tractography were determined. The following target and stimulation parameters were registered: active contacts, voltage, frequency, pulse width, and amplitude.

### 2.4. Data Analyses

Considering the small sample size and that data showed a non-normal distribution, non-parametric tests were run.

#### 2.4.1. Primary Outcome: change in BMI Value 

To test the effects of DBS on the primary outcome measure (BMI), Friedman’s non-parametric, repeated-measures analysis of variance (ANOVA) was used to compare BMI values at surgery to BMI values at each of the six-monthly postoperative determinations. As the main aim of the study was to evaluate treatment effects after 6 months of DBS, the Wilcoxon rank test was also used to compare two data points: pre-surgery BMI versus BMI at month 6.

Some participants required inpatient care before surgery to reach the minimum preoperative BMI value. Consequently, this preoperative BMI was not their “usual” BMI, but rather the result of inpatient treatment. To reflect this, we determined a BMI reference value (BMI-RV) for each patient, which was calculated differently depending on whether or not the patient required preoperative inpatient care. For patients who required preoperative inpatient care (patients 3, 4, 6 and 8), the BMI-RV was defined as the mean BMI achieved in the year prior to surgery (not including the BMI values obtained during inpatient care). For patients that did not require preoperative inpatient care (patients 1, 2, 5, 7), the BMI-RV was defined as the mean BMI achieved in the three-month period immediately prior to surgery. The same statistical analyses described above (Friedman’s and Wilcoxon) were repeated, but this time based on the BMI-RV values. The BMI-RV value was used to determine treatment response, which was defined as an increase of ≥10% in the pre-treatment BMI-RV value.

For exploratory purposes, the sample was also divided into different subgroups (i.e., participants who received preoperative inpatient care versus those that did not and participants with SCC stimulation versus participants with NAcc stimulation) to test for possible changes in BMI values between those groups (Wilcoxon rank test).

#### 2.4.2. Secondary Outcomes: Change in Anorexia Nervosa Behavior and Clinical Variables

Given the small sample size, the data on AN-related behaviors are given only as descriptive data. The Wilcoxon signed-rank test was used to compare scores obtained on the various instruments administered at baseline with the scores obtained at the end of month six of follow up. The results of this pre-post comparison were also used to determine whether participants who received DBS to the SCC or NAcc differed in terms of their relative improvement on depression and obsessive-compulsive scores (HAMD-17 and YBOCS, respectively).

## 3. Results

### 3.1. Participants Characteristics 

The study sample included eight participants (7 female), with a mean age at surgery of 40.75 years (standard deviation (SD) = 15.49). Most patients *(n* = 6) had a primary diagnosis of AN-restrictive type. The mean time since disease onset was 25.25 years (SD = 11.25). Comorbidities with other psychiatric diagnoses were frequent, most commonly major depressive disorder (MDD, *n* = 7), followed by panic disorder (PD, *n* = 3), and obsessive-compulsive disorder (OCD, *n* = 3). Six of the eight patients (75%) were taking benzodiazepines, while three were under antidepressant treatment, and one was taking antipsychotic medication.

The minimum and maximum BMI values over the last five years were registered. We evaluated variability in the BMI values over the 15 months prior to DBS implantation. The patients presented three main BMI fluctuation patterns over this period, which we classified as pattern A, B, or C. Pattern A consisted of a stable BMI trajectory, observed in participants 1, 4, 5, 7, and 8. Pattern B was characterized by frequent hospital admissions and ascendant and descendent peaks in BMI (unstable BMI trajectory), which was observed in two patients (3 and 6). Finally, one patient (2) showed a stable but descendent weight trajectory, which was denominated pattern C. Preoperative inpatient care was required in four cases to achieve the minimum BMI (=13) level required for surgery. Of these four patients, only one (participant 4) did not achieve the minimum BMI; however, the patient’s BMI (12.12) was considered acceptable and surgery was performed.

In participants 1, 2, 3, and 4, target selection was based on anatomical/stereotactic references; in participants 5, 6, 7, and 8, target selection was based on anatomical/stereotactic references as well as DTI data. Stimulation started at 3.5 MA for all patients and was maintained or increased accordingly to patient response, which was assessed monthly. The maximum stimulation was set at 8 MA.

**Figure 2:**
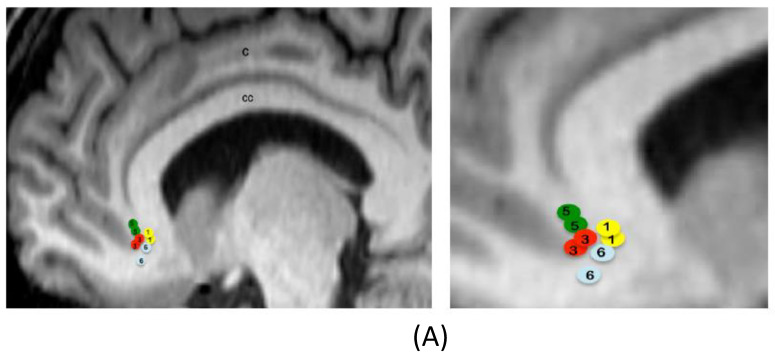

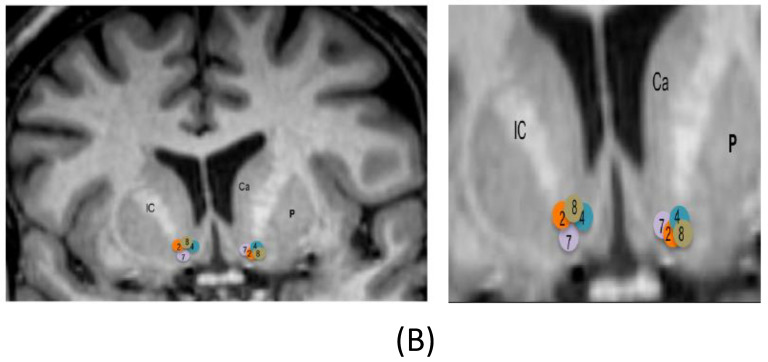
Location of electrode active contacts. (**A**) Location of electrode contacts on a sagittal view for patients with DBS on the subcallosal cingulate. Circles are schematic representations of electrode active contacts. Numbers within circles correspond to each patient. The figure at the right side is an enlargement. C = cingulate. CC = corpus callosum. (**B**) Location of electrode contacts on a coronal view for patients with DBS on the nucleus accumbens. Circles are schematic representations of electrode active contacts. Numbers within circles correspond to each patient. The figure at the right side is an enlargement. CA = caudate nucleus. CI = internal capsule. P = putamen. DBS = deep brain stimulation.

Table 1 describes the demographic and clinical characteristics of the sample, with the active contacts and stimulation parameters. Figure 2 shows location of electrode active contacts.

### 3.2. Primary Outcome: Change in Body Mass Index Values

**Figure 3:**
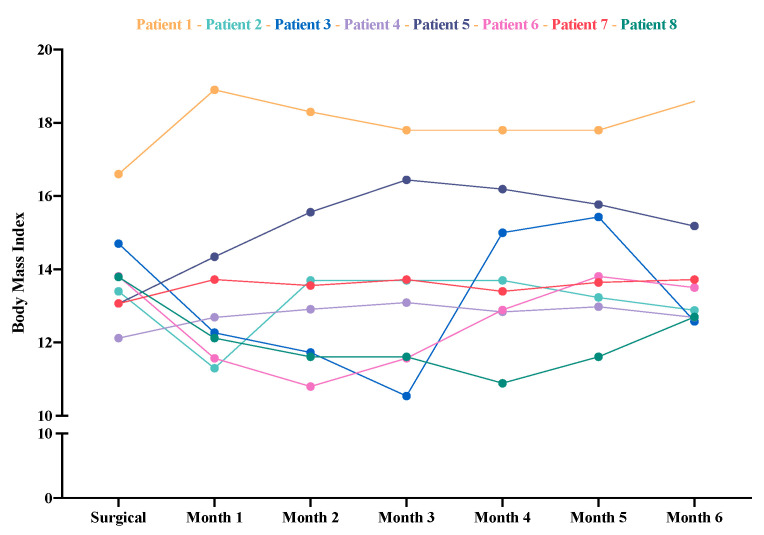
Individual changes in body mass index (BMI) during the six month follow-up period. BMI at surgery (criterion 1) was used as the reference value for this comparison.

To assess the effect of the DBS on BMI (the primary outcome measure), we compared the preoperative BMI for all patients (regardless of receiving inpatient care before surgery or not) to the BMI values measured at each postoperative time point (monthly). On this analysis, the Friedman repeated-measures ANOVA revealed no significant increase in BMI after surgery (*X^2^* = 2.71, *p* = 0.84). A Wilcoxon signed-rank test was performed to compare only two data points: preoperative BMI with BMI at month 6 of follow up, also revealing no significant changes (*Z* = –0.28, *p* = 0.78). Figure 3 shows the mean BMI scores for each patient over the six-month study period.

These analyses were repeated using a BMI reference value (BMI-RV; see the Data Analyses Section for a description) for each patient as the pre-operative BMI measure. The Friedman repeated measures ANOVA showed no significant increase in BMI (*X^2^* = 7.96, *p* = 0.24). However, when we compared only two data points (BMI-RV with the BMI obtained at the six month follow up), the increase was significant (BMI-RV: M = 12.67, SD = 1.64 versus BMI value at month 6: M = 13.98, SD = 2.05, *Z* = –2.38, *p* = 0.02).

The data were analyzed again but using a different approach (as explained above), in which treatment response was defined as a ≥10% increase in BMI-RV. Patient 1 showed a sustained gain in BMI (10%) at all monthly time points throughout the six month follow up. By contrast, patients 2 and 8 did not achieve a 10% gain in BMI at any time point. Finally, the other three patients showed a variable pattern during the follow-up period. However, at month 6, five of the eight participants presented an increase of at least 10% in BMI. Table 2 shows the reference BMI values for each patient and their response.

Lastly, were performed other analyses (Wilcoxon rank test) to explore differences between the subgroups. No significant pre/post (6 months) differences in BMI were found for participants who received preoperative inpatient care (*Z* = −1.09, *p* = 0.27) versus those who did not (*Z* = −1.46, *p* = 0.14). Similarly, neither subgroup (SCC nor NAcc) showed a significant increase in BMI: SCC stimulation (*Z* = 0.00, *p* = 1.00) versus NAcc stimulation (*Z* = 0.00, *p* = 1.0).

### 3.3. Secondary Outcomes: Change in Anorexia Nervosa Behaviors and Clinical Variables 

Patient 1 showed a reduction in daily physical activity (walking), which decreased from 6 hours per day to one hour/day at the one-month follow up. This reduction was maintained over the 6-month study period. Patient 2 also reduced the amount of daily physical activity from 6 hours to 3. Two patients (4 and 8) presented purging behavior prior to DBS. During the five years prior to DBS, patient 4 had maintained an exclusively liquid diet. After one month of DBS, the patient included two solid meals a day. Diuretic/laxative intake (patient 4) was significantly reduced from 40 tablets of furosemide a day to 5 tablets a day, and from 70 powder laxative sachets at baseline to complete abstinence. For patient 8, the purging frequency remained unchanged at month 6.

**Figure 4:**
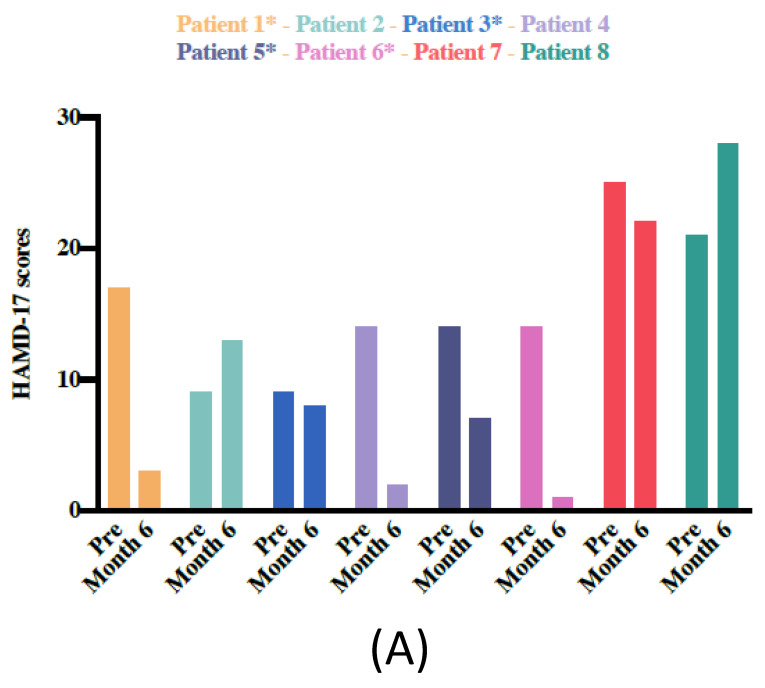

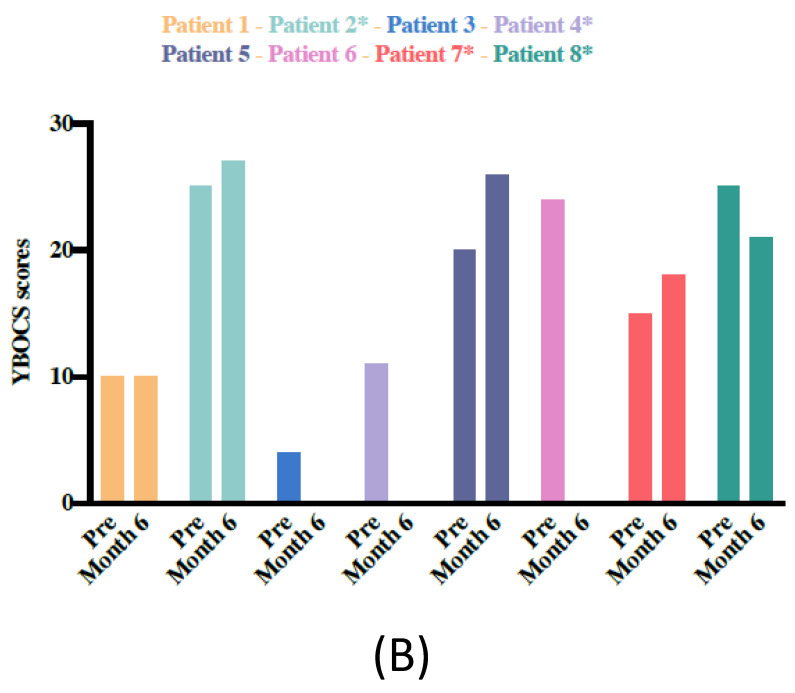
Changes in depression (HAMD-17) and obsessive-compulsive (YBOCS) scores for each patient. (**A**) Changes in depression scores based on the Hamilton Rating Scale for Depression (HAMD-17) from baseline to month 6 post-DBS. Patients identified with an asterisk (*) are those whose main comorbidity was an affective disorder, with SCC stimulation. (**B**) Changes in obsessive-compulsive symptoms based on the Yale-Brown Obsessive-Compulsive Scale (YBOCS) from baseline to month 6 post-DBS. Patients identified with an asterisk (*) are those whose main comorbidity was an anxiety disorder, with NAcc stimulation. HAMD-17 = Hamilton Depression Rating Scale. YBOCS = Yale-Brown Obsessive-Compulsive Scale.

No significant improvement was observed (Wilcoxon signed-rank test) for most clinical variables. However, a significant change was found in SF-36 scores at month 6, indicating an improvement in the patients’ quality of life (*Z* = 2.10, *p* = 0.03). Patients stimulated at the SCC (patients: 2, 4, 7, 8) whose predominant comorbidities were affective disorders presented larger improvements in depression scores (HAMD-17) than patients with SCC stimulation (SCC target: M_pre_ = 13.50, SD = 3.31 versus M_6months_ = 4.75, SD = 3.30, *Z* = −1.82, *p* = 0.06; NAcc target: M_pre_ = 17.25, SD = 7.13; M_6months_ = 16.25, SD = 11.32, *Z* = 0.00, *p* = 1.00); however, these differences were not statistically significant. In terms of YBOCs scores, no significant differences were found for patients with SCC stimulation or those who received NAcc stimulation (SCC target: M_pre_ = 18.50, SD = 6.60; M_6months_ = 16.50, SD = 11.61, *Z* = –0.55, *p* = 0.58; NAcc target: M_pre_ = 14.50, SD = 9.14 versus M_6months_ = 9.00, SD = 12.27, *Z* = 0.53, *p* = 0.59). Table 3 shows the results of the secondary outcomes from baseline to the 6 months follow-up assessment. Figure 4A, B shows changes in depression (HAMD-17) and obsessive-compulsive (YBOCS) scores for each patient.

### 3.4. Adverse Events

Cutaneous complications occurred in 3 patients (2, 5, and 6). Approximately 72 h after the procedure, patient 2 developed a greyish coloration in the area of the right electrode, potentially indicative of reduced blood flow; three days later (day 6), a necrotic eschar was observed, requiring skin flap surgery. Ten days after surgery, patient 5 developed skin dehiscence at the site of the incision for the surgical fiducial marker. The dehiscence did not respond to conservative treatment, and therefore the affected area was surgically cleaned to prevent infection. Patient 6 developed a chronic infection at the site of the skin incisions for surgical fiducial markers. The infection did not respond to antibiotic treatment and surgical cleaning was performed. No other intraoperative or postoperative complications or adverse events were registered.

## 4. Discussion 

Overall, the initial results of this study of six months of DBS to the NAcc and SCC in patients with severe and chronic AN show that DBS did not produce a statistically significant increase in BMI. Five out of eight patients achieved an increase of ≥10% in BMI (at month 6), and three out of eight presented changes in AN behaviors, including reduced physical activity and use of laxatives and diuretics. At month 6, DBS was associated with improvements in a patient-reported measure of quality of life (SF-36). Almost 40% of the patients treated developed skin complications that required treatment, including surgery.

As indicate above (in the Methods Section), we used two different criteria to assess the primary outcome (change in BMI). First, we compared preoperative BMI to postoperative BMI values obtained at the monthly assessments, finding no significant increase in body mass in the overall sample. This lack of a significant difference in BMI could be attributed to the particular characteristics of our patient sample versus other studies that did find significant differences in BMI after DBS [29,30,32,34]. First, unlike many of those studies, we included treatment-resistant, chronic patients. Second, we required a minimal preoperative BMI (13), which meant that half of the patients (*n* = 4) required inpatient intensive treatment to gain weight before they could undergo DBS implantation. Although we managed to recruit the sample over a one-year period, many patients with a severe AN profile declined to participate in the study due to this criterion (i.e., they were unwilling to gain weight). For some patients with AN, the idea of participating in a clinical trial whose main aim is to restore weight can seem to contradict their personal objectives. Third, we used different stimulation targets (SCC or NAcc) depending on the comorbid psychopathology (affective predominance or anxious predominance, respectively). Previous studies [29,31,32,33,34] have also evaluated DBS for AN, using the same targets (SCC and NAcc). However, to our knowledge, none of those studies considered comorbidities or the type of AN when selecting the DBS target. This is relevant given that data obtained from studies using functional MRI reveal differences between AN subtype (restrictive versus bingeing/purging) in terms of brain activation [12,14]. Moreover, in contrast to our study, most patients included in previous studies were characterized by less severe AN with a shorter time from diagnosis and/or were willing to achieve a BMI > 15 before undergoing surgery [30,31,36].

Interestingly, when we calculated a preoperative reference BMI value (BMI-RV) for each patient, we found a significant increase in BMI at month 6 (versus the preoperative BMI-RV), although the mean BMI at this follow-up assessment was still quite low (M = 13.98). Although the use of this novel value (BMI-RV) could be questioned, we believe that it better captures the patients’ true BMI before surgery, as it reflects the mean BMI over a longer time frame beyond just the immediate preoperative period.

When we performed another analysis in which treatment response was defined as an increase in BMI ≥ 10% (considered sufficient in this group of chronic, severe patients), five of the eight participants met this objective at month 6 and were thus considered responders (Table 2). Using the 10% gain in BMI as the cut-off to define treatment response, we observed different patterns. Of the four patients who met this criterion and were thus considered responders (patients 1, 3, 5, and 7), three received DBS stimulation to the SCC. In addition, most of these patients (1,5, and 7) presented a pattern A BMI trajectory before surgery, while only one patient (3) was characterized in the “unstable BMI trajectory” group (pattern B). Patient 2 showed a clear pattern of no-response that was consistent with the preoperative BMI pattern (sustained disease severity with clinical worsening). Together, these findings seem to suggest a link between the BMI pattern (i.e., illness trajectory) and the impact of DBS; however, more data are needed to corroborate this potential association. AN-specific behaviors in our sample were inverse, as some patients (1, 2, 4) presented a decrease in physical activity and diuretic/laxative use.

Of the various secondary clinical outcomes evaluated in this study, most did not show statistically significant improvements, which could be due to the short follow-up [34]. However, two findings were particularly noteworthy. First, we observed a significant increase in SF-36 scores, which indicates that the patients perceived a subjective improvement in quality of life after the intervention (month 6). Second, as we expected, patients whose predominant comorbidities were affective disorders and received stimulation to the SCC presented larger improvements in depression scores, while those with NAcc stimulation presented a greater improvement in the YBOCS (although none reached statistical significance). However, a positive impact on these measures related to features of AN (YBC-EDS; MAIA and Gardner) was not evidenced, in contrast to the findings of Lipsman et al. [34], who reported significant reductions on several subscales of the YBC-EDS. Nevertheless, the results of our study and those of Lipsman and colleagues [34] are not directly comparable, as that study had a much longer (12 months) follow-up period.

These results must be interpreted in the context of the study limitations. First, the small sample size and preliminary findings. Nevertheless, our data were obtained from a real-world sample of patients with chronic AN, a population in urgent need of novel treatment options such as DBS. Second, a recommended cut-off point for inclusion in this trial was BMI ≥ 13. To reach this cut-off point, some participants required inpatient intensive care, and therefore, the BMI reached at pre-surgery reflected this time under treatment. This cut-off point was established because excessively thin patients are more likely to develop pressure ulcers caused by the pulse generator implanted under the skin (due to the decreased skin thickness and greater fragility of the subcutaneous tissue). Although a higher BMI cut-off point would reduce this risk even further, doing so would likely require the exclusion of very severe patients (which is why we offered inpatient treatment to raise the BMI in selected patients). Indeed, even though we provided preoperative inpatient care, one patient still underwent surgery despite not reaching the minimum BMI. The decision to allow this patient to participate was made after careful consideration. The research team concluded that the patient’s safety could be ensured and therefore we decided to proceed. The patient did not experience any adverse events and, despite a lack of response in terms of BMI, her use of diuretics/laxatives decreased significantly, which we consider a good outcome. A third limitation is that we only included one male. Consequently, it was not possible to assess differences between men and women in treatment response. Fourth, the relatively short follow-up (6 months) is a limitation; however, the longer-term stability of our findings will be reported when data from the phase III part of this trial (12 months of follow up) become available. In addition, we observed cutaneous complications, which might be related to lower BMI and to other differences in the patients’ characteristics. In some patients, resolution of the cutaneous complications required surgery, as less invasive treatments were unsuccessful. However, and in contrast with other studies, no devices needed to be explanted.

## 5. Conclusions

After 6 months of DBS, some patients in this study with severe, chronic AN showed some benefits: increase in BMI, reduction in AN behavior, and improvement in quality of life (regardless of whether or not BMI improved). The percentage of patients developing cutaneous complications was high, but effectively resolved. Due to the short follow-up (6 months), we cannot reach any conclusions regarding the superiority of the target site (NAcc versus SCC) in terms of treatment outcomes. However, in the future, we will report longer term outcomes (12 months), which will provide a clearer picture of the long-term stability of BMI. Studies that include larger samples are needed to clarify whether DBS in patients with AN is associated with an improvement in comorbid symptoms (i.e., depression, anxiety). Finally, more research is needed to better characterize the relationship between BMI fluctuations, comorbid symptoms, and DBS targets.

## Figures and Tables

**Table 1:** Participant’s demographic and clinical characteristics, active contacts, and stimulating parameters.

Patient	Sex	Age	Type AN	AN Duration (Years)	Inpatient Care before Surgery	Minimum BMI	Maximum BMI	Reference BMI Value	Pharmacological Treatment	Main Comorbidity	Target	Active Contact (−), left	Active Contact (−), right	Maximum Intensity (MA)
1	Female	37	Restrictive	26	NO	14.64	16.22	16.22	Clonazepam	Affective Disorder	SCC	2	2	7
2	Male	45	Restrictive	32	NO	13.44	17.51	13.44	None	Anxiety Disorder	NAcc	1	1	8
3	Female	45	Restrictive	16	YES	10.06	11.72	10.94	Citalopram, Diazepam, Olanzapine, Lormetazepam	Affective Disorder	SCC	3	1	8
4	Female	39	Purgative	25	YES	11.47	12.55	11.83	Lorazepam	Anxiety Disorder	NAcc	2	1	8
5	Female	36	Restrictive	22	NO	13.07	15.18	13.07	Sertraline, Venlafaxine, Mirtazapine	Affective Disorder	SCC	2.3	2	7
6	Female	33	Restrictive	21	YES	9.64	12.73	11.57	Bromazepam	Affective Disorder	SCC	1.2	3.4	8
7	Female	57	Restrictive	41	NO	11.92	12.74	12.33	Venlafaxine, Mirtazapine, Bromazepam, Lormetazepam	Anxiety Disorder	NAcc	2	2.3	7.5
8	Female	34	Binge-Purge	19	YES	11.61	12.34	11.98	Lorazepam	Anxiety Disorder	NAcc	3.4	3.4	8

*Note.* AN = Anorexia Nervosa. MINI = International Psychiatric Interview for Mental Disorders. BMI = Body Mass Index. OCD = Obsessive Compulsive Disorder. MDD = Major Depressive Disorder. SCC = subcallosal cingulate. NAcc = Nucleus accumbens. MA = Milliampere. Minimum and maximum BMI refer to the last 5 years.

**Table 2:** Participant response defined as a 10% increase in BMI during the six-month follow-up period.

Patient	Reference BMI Value	Response Value (10% Increase)	Months						Response to DBS (YES/NO)
			**1**	**2**	3	4	5	6	
1	16.22	17.842							YES
2	13.44	14.784							NO
3	10.94	12.034							YES
4	11.83	13.013							NO
5	13.07	14.377							YES
6	11.57	12.727							NO
7	12.33	13.563							YES
8	11.98	13.178							NO

*Note.* A green box indicates that the patient achieved a 10% increase in BMI at a given month while a red box means that the patient did not reach the 10% BMI gain threshold for that month. DBS = deep brain stimulation. BMI = body mass index.

**Table 3:** Wilcoxon signed-rank test for secondary outcomes measured preoperatively (baseline) and at month 6 of follow up.

	Mean	Standard Deviation	Z	*p*
**HAMD-17**				
Pre-surgery	15.38	5.52	−1.47	0.14
Month 6	10.50	9.87		
**YBOCS**				
Pre-surgery	16.50	7.69	−0.85	0.39
Month 6	12.75	11.76		
**HAM-A**				
Pre-surgery	13.63	6.30	−1.26	0.21
Month 6	10.94	11.84		
**YBC-EDS**				
Pre-surgery	111.38	48.28	−1.54	0.12
Month 6	87.62	64.26		
**MAIA**				
Pre-surgery	15.53	7.11	0.42	0.67
Month 6	15.15	7.61		
**Gardner—Distortion**				
Pre-surgery	2.50	3.65	−0.70	0.48
Month 6	2.75	4.09		
**Gardner—Dissatisfaction**				
Pre-surgery	2.75	3.19	−0.32	0.74
Month 6	3.00	4.17		
**SF36**				
Pre-surgery	32.18	16.98	2.10	0.03
Month 6	60.56	22.40		
**BIS-11**				
Pre-surgery	43.88	19.66	−0.42	0.67
Month 6	42.25	10.06		

*Note.* HAMD-17 = Hamilton Depression Rating Scale. YBOCS = Yale-Brown Obsessive-Compulsive Scale. HAMA = Hamilton Anxiety Rating Scale. YBC-EDS = Yale-Brown-Cornell Eating Disorders Scale. MAIA = Multidimensional Assessment of Interoceptive Awareness. Gardner Assessment of Body-Image. SF36 = Short Form Health Survey. BIS-11 = Barrat Impulsivity Scale 11.
